# Sustained Euthyroidism during Thionamide Antithyroid Drug Treatment: Comparison of Short- and Long-term Methimazole Therapy

**DOI:** 10.5812/ijem-140956

**Published:** 2024-01-02

**Authors:** Navid Saadat, Safdar Masoumi, Mohammad Karim Shahrzad, Fereidoun Azizi

**Affiliations:** 1 Prevention of Metabolic Disorders Research Center, Research Institute for Endocrine Sciences, Shahid Beheshti University of Medical Sciences, Tehran, Iran; 2 Endocrine Research Center, Research Institute for Endocrine Sciences, Shahid Beheshti University of Medical Sciences, Tehran, Iran; 3 Department of Biostatistics, Faculty of Medical Sciences, Tarbiat Modares University, Tehran, Iran; 4 Shohada Tajrish Medical Center, Shahid Beheshti University of Medical Sciences, Tehran, Iran

**Keywords:** Euthyroidism, Methimazole, Therapy, Thionamide, TSH Receptor Antibody

## Abstract

**Background:**

Extended low serum thyrotropin (TSH) levels may increase the risk of cardiovascular events in patients with hyperthyroidism.

**Objectives:**

This study aimed to compare the time spent with sustained normal TSH concentration following short- and long-term methimazole treatment.

**Methods:**

A total of 258 patients with Graves’ hyperthyroidism completed 18 - 24 months of methimazole therapy and were randomized to discontinue treatment (n = 128, short-term group) or continue an additional 36 - 102 months of methimazole therapy (n = 130, long-term group). Clinical and laboratory evaluations were performed every 6 months for 132 months after randomization.

**Results:**

There was no difference in serum-free thyroxine, triiodothyronine, and TSH concentrations between the 2 groups at the time of randomization. Of 128 patients in the short-term group, 5 left in follow-up, 2 became hypothyroid, 67 (54%) had a relapse of hyperthyroidism, and only 54 (44%) were euthyroid at the end of the study. Among 130 patients on the long-term methimazole therapy, 4 were left in follow-up, 24 developed hyperthyroidism, 4 developed hypothyroidism, and 98 (78%) were euthyroid 132 months post-randomization. Total time spent on euthyroidism was 90.4% ± 8.1% of the study period in the short-term and 95.8% ± 7.0% in the long-term treatment groups (P < 0.001). The lowest time spent in euthyroidism (74.6% ± 6.4% of the study period) belonged to 29 (24%) patients in the short-term group under levothyroxine therapy because of fluctuation in serum TSH. Patients in both groups with hyperthyroidism relapse who chose methimazole therapy spent >90% of the study time in euthyroidism.

**Conclusions:**

In patients with Graves' hyperthyroidism, sustained normal serum TSH levels were more common in the long term as compared to the short-term methimazole treatment.

## 1. Background

Graves’ disease is the most common cause of thyrotoxicosis. This autoimmune disease is caused by thyrotropin (TSH) receptor antibodies (TRAb) stimulating the TSH receptor of the thyroid cell membrane. There are controversies in choosing the appropriate treatment for Graves’ disease. Radioactive iodine, antithyroid drugs (ATDs), and surgery are therapeutic modalities for diffuse toxic goiter. All these approaches have advantages and disadvantages, and none of them can render all patients with Graves’ disease permanently euthyroid (1).

Thianomide antithyroid agents have been a mainstay in treating patients with Graves’ hyperthyroidism for over 75 years (2); however, the rate of relapse is approximately 50% following the cessation of 12 - 18 months of treatment. Recent reports indicate that long-term ATD (LT-ATD) therapy increases the remission rate up to 85% following the discontinuation of such treatment (3-5).

The main objective of hyperthyroidism treatment is to re-establish normal thyroid physiology (2). However, consideration of cardiovascular safety is of utmost importance when treating hyperthyroidism. An increase in all-cause mortality in patients with hyperthyroidism (6) raised the risk of mortality and substantial cardiovascular morbidity in patients with uncorrected hyperthyroidism (7). Moreover, the association of longer-suppressed serum TSH with cardiovascular outcomes in treated and untreated hyperthyroid patients (8) indicated that euthyroidism should be induced rapidly and sustained throughout the management of hyperthyroid patients (7, 9).

## 2. Objectives

The purpose of this study was to compare “the time spent in sustained euthyroidism” following short-term methimazole (ST-MMI) and long-term methimazole (LT-MMI) treatment in patients with Graves’ disease.

## 3. Methods

### 3.1. Study Design

In this study, we explored the follow-up data of one of the randomized control trials of towards outstanding hyperthyroid care induced by antithyroid drugs (TOHID) studies. TOHIDs registered in the Iranian Registry of Clinical Trials [https://irct.behdasht.gov.ir/trial/5143] are a combination of observational studies and clinical trials for evaluating treatment modalities in patients with hyperthyroidism (10, 11) in an iodine-sufficient region (12). The present clinical trial compared remission rates following the discontinuation of ST-MMI and LT-MMI treatments. Details of the trial were reported before (5). Briefly, 302 patients with an untreated first episode of Graves' hyperthyroidism were treated with MMI for a median of 19 months, and 258 of them were randomized to discontinue MMI (short-term group, n = 128) or continue methimazole for 36 - 120 months (long-term group, n = 130). Total time on methimazole was 60 - 120 months in the long-term group (mean: 95 ± 32 months). All patients were followed 132 months after randomization (Figute 1). 

**Figure 1.**
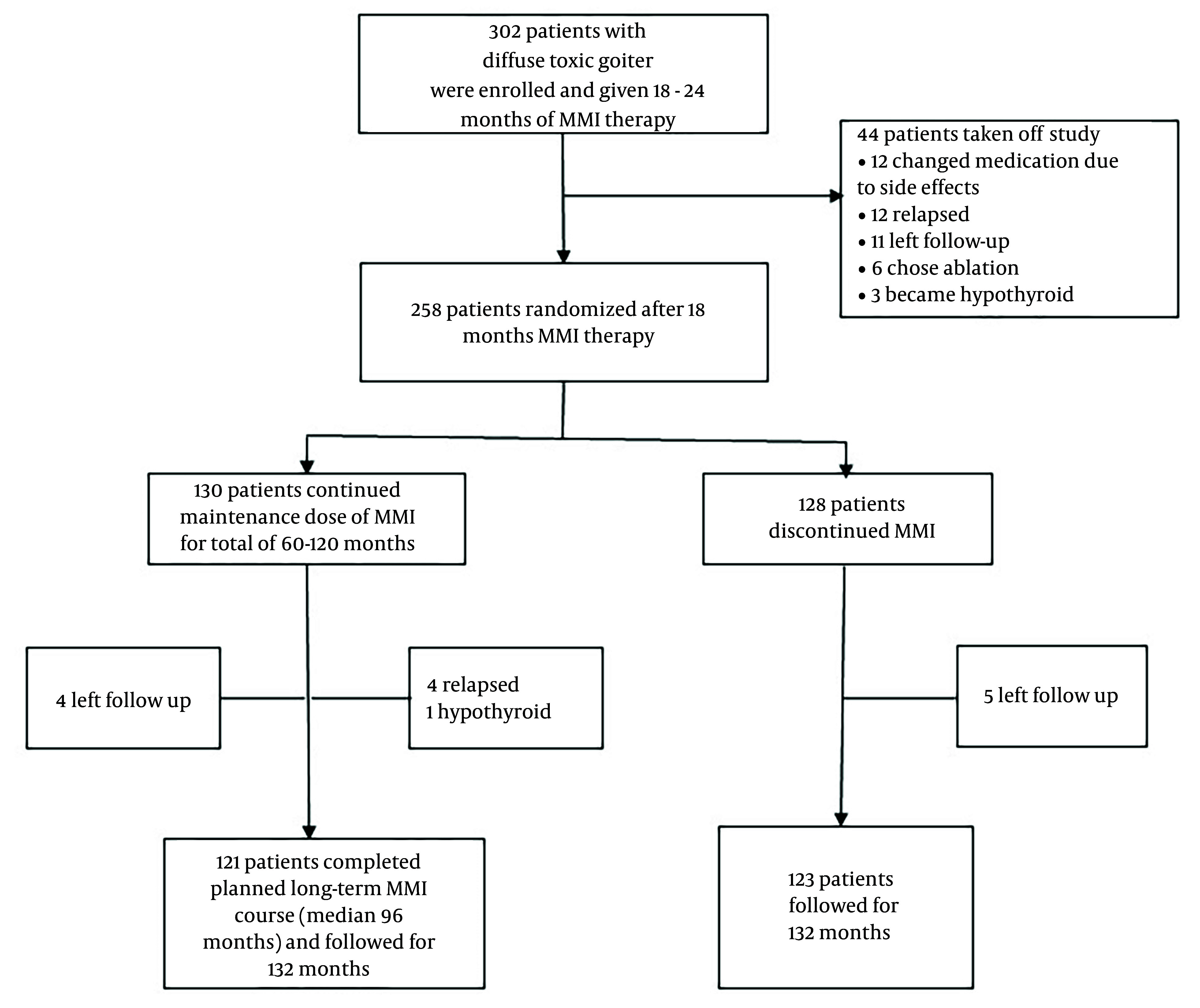
Study enrollment and follow-up for the recurrence of hyperthyroidism. MMI: methimazole.

### 3.2. Methimazole Therapy

After the daily administration of 20 - 30 mg MMI for one month, it was titrated to keep free thyroxine (FT4) between 9 - 23 pmol/L and TSH<5.09 mIU/L. Patients in the long-term group continued an additional course of methimazole after randomization, with further titration as needed to meet therapeutic goals.

### 3.3. Procedures

Demographic data were obtained, and goiter size was estimated with palpation at entry. Patients were followed every 6 months after randomization and at each visit. Serum concentrations of FT4, triiodothyronine (T3), TSH, and TRAb were measured by commercial kits. Inter-assay and intra-assay coefficients of variation were <6.1% and <9.1% for all measurements, respectively.

### 3.4. Endpoints

The primary endpoints were the percent of time spent in euthyroidism, hypothyroidism, and hyperthyroidism, as well as sustained euthyroidism from the day of randomization to the end of follow-up. Goiter was assessed by palpation according to the World Health Organization criteria (12).

### 3.5. Definitions

Overt hypothyroidism was defined as FT4<9 pmol/L and TSH>5.06 mIU/L for people in Tehran, Iran (13). Subclinical hypothyroidism was defined as TSH>5.06 mIU/L with normal serum FT4 and T3 concentrations. Hyperthyroidism was defined as TSH<0.4 mIU/L (reference range: 0.4-5.06 mIU/L) with FT4>23 pmol/L (reference range: 9 - 23 pmol/L) and/or T3>200 ng/dL (reference range: 75 - 200 ng/dL). Moreover, subclinical hyperthyroidism was defined as TSH<0.4 mIU/L with serum FT4 and T3 within the reference ranges.

### 3.6. Statistical Analysis

Two hundred sixty patients were needed for a small-medium effect size of 0.40, with 80% power and 5% significance, allowing for 30% attrition. Two hundred forty-two patients completed the study. The post-hoc test power for this number of patients was calculated with the same effect of 87%.

All analyses were performed using IBM SPSS statistical software version 25 (SPSS Inc., Chicago, IL, USA). Quantitative variables were summarized using mean and standard deviation. Qualitative variables were reported as frequency and percentage. Variables were compared between two groups with Student t, Mann-Whitney, chi-square, or Fisher’s exact tests. Time spent on various thyroid function statuses for each patient was calculated from the date of randomization when all patients in both groups were euthyroid. Percent of time that serum TSH, FT4, and T3 remained in various thyroid function categories, according to the definitions mentioned above, was calculated. The event date for thyroid status cases was described as the middle time between the date of the follow-up visit and the diagnosis. Two-tailed P-value<0.05 was considered significant.

## 4. Results

### 4.1. Patient Characteristics

A total of 258 patients completed 19 ± 3 months of methimazole treatment before randomization. All patients had normal serum FT4, T3, and TSH concentrations at randomization (Table 1). 

**Table 1.** Patient Characteristics by Study Arms at the Time of Randomization ^a^

Variables	Short-term Methimazole (n = 128)	Long-term Methimazole (n = 130)
**Age (y)**	39.7 ± 12.6	42.8 ± 13.3
**Female**	94 (73)	104 (80)
**Goiter grade**		
0 or 1	61 (48)	96 (74)
2	67 (52)	34 (26)
**Current smoking**	20 (16)	17 (13)
**FT4 (pmol/L)**	16.9 ± 3.2	16.7 ± 2.5
**T3 (ng/dL)**	130 ± 21	133 ± 19
**TSH (mIU/L)**	1.7 ± 1.2	2.1 ± 1.8
**TRAb (IU/L)**	1.0 ± 0.7	1.2 ± 0.9

Abbreviations: FT4, free thyroxine; T3, triiodothyronine; TSH, thyrotropin; TRAb, thyroid receptor antibodies.

^a^ Values are expressed as mean ± SD or No. (%).

Among 258 randomized patients, 130 were assigned to longer-term therapy, and 128 discontinued methimazole (short-term group). Of these 128 patients, 5 were left in follow-up. By the end of 132 months post-randomization, 67 (54%) patients had a relapse of hyperthyroidism, and 2 patients developed hypothyroidism. Therefore, only 54 (44%) cases were euthyroid at the end of the study without receiving treatment for thyroid dysfunction. Out of 130 patients on long-term methimazole therapy, 4 were left to follow up, 24 developed hyperthyroidism, 4 developed hypothyroidism, and 98 (78%) were euthyroid at 132 months post-randomization (Table 2). 

**Table 2.** Thyroid Function Status During Post-Randomization 132 Months of Follow-up ^a^

Variables	Short-term Methimazole (n = 128)	Long-term Methimazole (n = 130)
**Left follow-up**	5	4
**Thyroid status outcome**		
Relapse of hyperthyroidism	67 (54)	24 (19)
Spontaneous hypothyroidism	2 (2)	4 (3)
Euthyroidism	54 (44)	98 (78)
**Thyroid status at the end of the study**		
Euthyroid	74 (60.1)	98 (77.7)
Hypothyroid on levothyroxine	29 (23.6)	7 (5.6)
Hyperthyroid on methimazole	20 (16.3)	21 (16.7)

^a^ Values are expressed as No. (%)

### 4.2. Thyroid Status at the End of the Study

Of patients who had a hyperthyroidism relapse during the study period, 46 and 2 chose radioactive iodine, while 21 and 22 patients chose long-term methimazole treatment in the short- and long-term groups, respectively. We observed that 28 of 48 patients treated with radioactive iodine became hypothyroid, and 20 became euthyroid. Among 43 patients on long-term methimazole, 41 were euthyroid, and 2 were hypothyroid by the end of the study (Figure 1). 

### 4.3. Time Spent on Various Thyroid Status

Patients in the short- and long-term methimazole groups spent 90.4% ± 8.1% and 95.8% ± 7.0% of the study period in euthyroidism, respectively (P < 0.001). The time spent in euthyroidism was not significantly different between the two groups: 94.5 ± 5.1 vs. 95.8 ± 7.0 months, respectively. The lowest time spent in euthyroidism (74.6 ± 6.4 months) belonged to 29 (24%) of the patients in the short-term group who were on levothyroxine treatment because of hypothyroidism. Patients in both groups who had a recurrence of hyperthyroidism and chose methimazole therapy spent >90% of their study time in euthyroidism (Table 3). 

**Table 3.** Percent of Time Spent in Various Thyroid Function Statuses in Patients with Graves Hyperthyroidism Treated with Short- and Long-Term Methimazole ^a^

Methimazole Treatment Group	Percent of Time Spent in		
	Euthyroidism	Subclinical Hyperthyroidism	Subclinical Hypothyroidism
**Short-term (n = 123)**	90.4 ± 8.2 ^b^	6.7 ± 5.8	2.9 ± 4.0
Euthyroid (74)	94.5 ± 5.1	3.5 ± 3.9	2.0 ± 3.6
Hypothyroid on levothyroxine (29)	79.6 ± 6.4 ^b^	13.5 ± 4.9	6.9 ± 3.4 ^b^
Hyperthyroid on methimazole (20)	91.8 ± 5.2	7.8 ± 5.4	0.4 ± 0.1
**Long-term (n = 126)**	95.0 ± 7.1	4.8 ± 7.2	0.2 ± 0.9
Euthyroid (98)	95.8 ± 7.0	4.1 ± 6.6	0.1 ± 0.8
Hypothyroid on levothyroxine (7)	92.3 ± 10.8	7.7 ± 10.8	0
Hyperthyroid on methimazole (21)	91.5 ± 7.7	8.2 ± 8.0	0.3 ± 1.1

^a^ Values are expressed as mean ± SD.

^b^ P < 0.001, compared to the long-term methimazole group.

### 4.4. Changes in Body Weight

There was a 1.45 ± 3.65 and 0.3 ± 3.5 kg increase in the body weight in the short- and long-term groups, respectively (P = 0.017).

### 4.5. Adverse Events

During short-term MMI treatment, minor complications, such as cutaneous reactions, leukopenia, elevated liver enzymes, and arthralgia, occurred in 12% of patients. No case of major adverse events was observed. After one year of MMI therapy, there was no case of minor or major complications.

## 5. Discussion

The present study reports extended follow-up of thyroid status following short- and long-term continuous methimazole therapy. The results showed that those treated with long-term methimazole spent more time in euthyroidism during the 132-month post-randomization period.

Sustained normal levels of TSH during the post-treatment follow-up of patients with hyperthyroidism are an essential aspect of cardiovascular safety. All-cause mortality may increase in hyperthyroidism due to both nodular and diffuse toxic goiter (6). If thyrotoxicosis is not adequately corrected, it may be accompanied by an augmented risk of mortality, stroke, health failure, and cardiac arrhythmias (7). It has been reported that suppressed TSH for a long duration in both treated and untreated patients is associated with a rise in cardiovascular outcomes (8). Early and effective control of hyperthyroidism, regardless of treatment modality, is associated with better-improved survival than less effective control of the disease (7). Therefore, the main objective in managing patients with hyperthyroidism should focus on inducing rapid euthyroidism and its maintenance throughout management (7, 9).

It has been reported that after conventional 12 - 18 months of ATD treatment, 53% of patients with TRAb titers of normal or slightly elevated 0.9 - 4.4 IU/L at the end of therapy experience hyperthyroidism relapse after the cessation of ATD therapy (14). Fluctuating TRAb titers probably cause relapse in 30% - 40% of Graves’ patients during the first 5 - 6 years of ATD therapy (15, 16). Few studies have shown that remission rates after ATD treatment up to 42 months were not significantly different from ATD therapy for 12 - 24 months (17-20). Therefore, the length of long-term therapy for optimum TRAb suppression and the highest remission rate has been suggested at ≥60 months of treatment (5, 21).

The findings of the present study show that the time spent in euthyroidism during the years of continuous methimazole is longer when the original course of treatment is ≥60 months compared to a conventional shorter treatment period of 18 months. The difference is mainly due to the lower recurrence rate of hyperthyroid patients who have long-term compared to short-term methimazole treatment (18% vs. 50% in the percent study), among those who had a recurrence of thyrotoxicosis, treatment with additional courses of methimazole induced longer euthyroidism compared to patients who chose radioiodine treatment because most of such patients develop hypothyroidism and require life-long levothyroxine therapy. Considerable variability of serum TSH during levothyroxine treatment has been reported by many studies, causing periods of subclinical hyper- and hypothyroidism (22-24).

The strength of this study is that we aimed, designed, and performed the study to address a knowledge gap in the comparative effectiveness of short-term versus long-term methimazole treatment duration regarding the induction of sustained euthyroidism. The findings should aid decision-making in adapting the most appropriate therapy for patients with hyperthyroidism.

This study has some limitations. First, the survey was not double-blinded and may contain selection and assignment biases. The goiter size at the time of randomization was smaller in the long-term group than in the short-term group; however, thyroid function tests and TRAb values did not differ between the two groups. Second, the number of visits and frequency of laboratory assessments were limited; therefore, the exact time of sustained euthyroidism may be debatable. Third, patients' quality of life, as an essential outcome, was not studied in patients of the two groups.

### 5.1. Conclusions

Long-term methimazole therapy is accompanied by more sustainable normal serum TSH during long-term follow-up than short-term (conventional) methimazole therapy. This additional benefit, along with the cure of hyperthyroidism in approximately four-fifths of patients after an extended follow-up of 132 months, indicates the superiority of long-term versus conventional short-term methimazole treatment.

## Clinical Trial Registration Code:

IRCT201009224794N1.
